# Microbial feed additives in ruminant feeding

**DOI:** 10.3934/microbiol.2024026

**Published:** 2024-07-11

**Authors:** Ahmed E. Kholif, Anuoluwapo Anele, Uchenna Y. Anele

**Affiliations:** 1 Department of Animal Sciences, North Carolina Agricultural and Technical State University, Greensboro, NC 27411, USA; 2 Dairy Science Department, National Research Centre, 33 Bohouth St. Dokki, Giza, Egypt; 3 Department of Natural Resources and Environmental Design, North Carolina Agricultural and Technical State University, Greensboro, NC 27411, USA

**Keywords:** Direct feed microbial, feed additives, microorganisms, mode of action, performance, ruminants

## Abstract

The main purposes of feed additives administration are to increase feed quality, feed utilization, and the performance and health of animals. For many years, antibiotic-based feed additives showed promising results; however, their administration in animal feeds has been banned due to some public concerns regarding their residues in the produced milk and meat from treated animals. Some microorganisms have desirable properties and elicit certain effects, which makes them potential alternatives to antibiotics to enhance intestinal health and ruminal fermentation. The commonly evaluated microorganisms are some species of bacteria and yeasts. Supplementing microorganisms to ruminants boosts animal health, feed digestion, ruminal fermentation, animal performance (meat and milk), and feed efficiency. Moreover, feeding microorganisms helps young calves adapt quickly to consume solid feed and prevents thriving populations of enteric pathogens in the gastrointestinal tract which cause diarrhea. *Lactobacillus*, *Streptococcus*, *Lactococcus*, *Bacillus*, *Enterococcus*, *Bifidobacterium*, *Saccharomyces cerevisiae*, and *Aspergillus oryzae* are the commonly used microbial feed additives in ruminant production. The response of feeding such microorganisms depends on many factors including the level of administration, diet fed to animal, physiological status of animal, and many other factors. However, the precise modes of action in which microbial feed additives improve nutrient utilization and livestock production are under study. Therefore, we aim to highlight some of the uses of microorganisms-based feed additives effects on animal production, the modes of action of microorganisms, and their potential use as an alternative to antibiotic feed additives.

## 1. Introduction

The world population is expected to exceed over 9.8 billion people by 2050, which is a great challenge for decision makers regarding food security. Livestock farming is one of the rapidly expanding agricultural sectors offering a sustainable solution to help in covering food requirements. Livestock production includes products (e.g., meat, milk, eggs, fish, etc.) from different species of farm animals. Therefore, improving productivity of animal per feed unit is a critical issue.

Ruminants digest feeds in its different parts (i.e., reticulum, rumen, omasum, and abomasum); however, the rumen is the fermentation vat of the fibrous feeds via ruminal microbiota [1]. However, improving the digestion and utilization of feeds is recommended. Therefore, animal microbiologists and nutritionists work hard to explore strategies to increase feed utilization, animal productivity, animal health, and enhance the safety of animal products. Their major strategies include facilitating optimal fermentation, reducing ruminal diseases, and eliminating pathogens. Some feed additives including antibiotics, probiotics, and prebiotics have been evaluated for their role in the rumen and other parts of the digestive tracts [2]. Microbial feed additives are mainly categorized into three main types including bacterial, fungal, and their mixture. The major functions of microbial feed additives (probiotics) are removing harmful microorganisms from the gastrointestinal tract and modifying the microbial population and promoting a more favorable balance between beneficial and harmful microorganisms [3]. Additionally, microbial feed additives are included in the diets of ruminants to improve feed digestion and modify the ruminal microbial profile, leading to better ruminal fermentation, enhanced feed utilization, and improved animal performance [3]–[5]. The commonly used microbial feed additives are *Lactobacillus*, *Streptococcus*, *Lactococcus*, *Bacillus*, *Enterococcus*, *Bifidobacterium*, and yeasts [6].

Using antibiotics as feed additives was prohibited due to antibiotic resistance in microbial populations causing some public concerns. In 2006, the EU Regulation (EC) No 1831/2003 banned the administration of antibiotics as feed additives. Therefore, administration of alternatives to antibiotics was recommended. Using microorganisms-based feed additives as potential alternatives to antibiotics gained increasing interests from animal nutritionists and microbiologists due to concerns over antibiotic resistance and the need for sustainable farming practices [7],[8]. Increasing antimicrobial resistance is a global health risk that requires an urgent attention [6]. Compared to antibiotic feed additives, probiotics can eradicate harmful microorganisms from the gastrointestinal tract and alter the host's microbial population density in the intestines. This leads to the establishment of a more favorable microbial community by shifting the balance between beneficial and harmful microorganisms [3]. In their review, Abd El-Hack et al. [5] stated that probiotics exhibit no adverse effects on animals, and they were tailored to specific strains of bacteria and demonstrated resistance to acid and bile. Probiotics exert their effects on pathogenic microbes through several primary mechanisms, including their competition with pathogenic bacteria by secreting substances like bacteriocins, organic acids, and hydrogen peroxide, which inhibit their growth, and competitively exclude pathogens by vying for nutrients and adhesion sites on the intestinal mucosa, thus preventing colonization [8]. Probiotics also compete for binding sites on the intestinal epithelium, thereby hindering pathogen attachment and enhancing nutrient utilization [9].

Animals begin to develop a symbiotic connection with endogenous microbiota to ferment plant cell wall polysaccharides that are resistant to mammalian enzymatic degradation after being fed microorganisms from exogenous sources [2],[4]. Some microorganisms have been evaluated as feed additives in diets of animals because they are cost-effective, stable, and could provide nutritional and functional benefits to animals. Feeding microorganisms to animals, which is known as direct feed microbial (DFM), is a tighter term than probiotics. Feeding microorganisms improves feed utilization and conversion by enhancing nutrient digestion and boosting nutrient use per unit of feed.

Microorganisms feed additives could be divided into groups with different characteristics and functions. Using microorganisms as feed additives has several advantages including: 1) The easy, applicable, and economical production of these products with high stability, 2) the ability to use microorganisms that are considered as co-products from the food industry, and 3) the high contents of nutrients, such as protein, amino acids, fats, and vitamins. However, some microorganisms have low digestibility, heavy metals, and toxicity [10].

Our main objective of this review is to evaluate the potential of microbial additives as substitutes for antibiotics in enhancing the growth efficiency and health of ruminants. In this review, we explored existing literature on feeding microbial-based feed additives to ruminant animals as potential alternatives to conventional antibiotics.

## 2. Direct-fed microbes

For many years, antimicrobial feed additives at sub-therapeutic dosages have been used to improve feed digestion and stimulate growth. However, the EU and many other countries banned the use of antimicrobial based feed additives due to some concerns about rising bacterial antibiotic resistance in people consuming animal products [11]. Therefore, exploring safe alternatives of antimicrobial feed additives is a critical issue for organic and sustainable livestock farming. Feeding live microorganisms (i.e., DFM and prebiotics) to ruminants is considered a safe strategy to achieve such purposes. Probiotic is a broad statement that refers to a variety of microbial cultures, extracts, and enzyme preparations. According to Elghandour et al. [4], ruminal probiotics may be defined as “live cultures of microorganisms that are intentionally introduced into the rumen to enhance animal health or nutrition”. DFM changes the microbial environment and fermentation properties for the better.

Anee et al. [3] summarized the basic modes of action of probiotic as: (1) Inhibition of pathogen adhesion; (2) production of antimicrobial components such as bacteriocins and defensins; (3) competitive exclusion of pathogenic microorganisms; (4) enhancement of barrier function; (5) reduction of luminal pH; and (6) modulation of the immune system.

As mentioned before, DFM may be divided into three categories, including bacterial, fungal, and their mixture:

### 2.1. Bacterial Direct-fed microbes

Bacteria are unicellular microorganisms with a size range of 0.5 to 5.0 µm [12]. The cell of bacteria is rich in lipids, proteins, and amino acids. Based on specific characteristics (i.e., cell wall structures), bacteria are categorized into gram-positive and gram-negative. The main component of bacterial cell wall is peptidoglycan, representing 40 to 60%, and is made of N-acetylglucosamine, N-acetylmuramic acid, and short peptide chains, including L-alanine, D-glutamic acid, either L-lysine or diaminopimelic acid, and D-alanine [13]. In the gram-positive bacteria, the cell wall is thicker because more peptidoglycan envelopes in the cell wall than gram-negative bacteria [14]. Moreover, the gram-negative bacteria have an outer membrane of lipopolysaccharide, and may be toxic and affect animal health [12],[15].

The bacterial DFM include mostly *Lactobacillus*, *Propionibacterium*, *Bifidobacterium*, *Enterococcus*, *Streptococcus*, *Bacillus*, *Megasphaera elsdenii*, and *Prevotella bryantii* [16]. Bacterial DFM may be divided into lactic acid-producing bacteria (LAB), lactic acid-utilizing bacteria (LUB), and other microorganisms (Table 1). Some criteria should be met to classify a microorganism as a microbial feed supplement including: the microbe must be non-pathogenic and non-toxic to the host animal, the microbe must be capable of producing antimicrobial agents, antagonistic toward pathogenic, the microbe must be able to adhere to and colonize the epithelial cells of the rumen and gut, the microbe must be capable of competing with normal microbiota and metabolizing in the gut environment (e.g., resistant to low pH, organic acids, bile salts, and digestive enzymes), and the microbe must be genetically stable [17].

**Table 1.** Common microorganisms used as microbial feed additives [18].

Product	Genus	Species	Respective effects	Reference
LAB	*Lactobacillus*	*L*. *acidophilus*	Reduces mortality. Improves growth. Improves immune defense mechanisms. Produces active dietary enzymes including protease amylase, lipase, phytase, and protease.	[19]–[22]
		*Lactiplantibacillus plantarum*		
		*Lacticaseibacillus casei*		
		*L*. *gallinarum*		
		*Ligilactobacillus salivarius*		
		*Limosilactobacillus reuteri*		
		*L. delbrueckii* subsp. *bulgaricus*		
	*Bifidobacterium*	*B. pseudolongum*	Maintains the balance of the intestinal microflora. Limits the risk of infections. Reduces coccidiosis in gut. Protective activity against Salmonella, Listeria and *E. coli*. Inhibits certain pathogens. Reduces diarrhea.	[22]–[24]
		*B. thermophilum*		
		*B. longum*		
		*B. lactis*		
	*Streptococcus*	*S. bovis*	Creates a healthier and more efficient digestive system. Improves fiber digestion. Stabilizes rumen pH. Enhances microbial populations. Improves production performance. Reduces pathogenic bacteria. Improves immune function.	[24]–[27]
		*S. faecium*		
	*Enterococcus*	*E. faecalis*	Breaks down the carbohydrates in the feed to produce lactic acid. Improves production performance. Improves animal immunity. Improves the intestinal environment.	[28]–[31]
		*E. faecium*		
		*E. faecal*		
LUB	*Megasphaera*	*M. elsdenii*	Improves ruminant performance. A causative agent of milk fat depression in high-producing dairy cows. Stimulates ruminal development in pre-weaning animals. Prevents milk fat depression. Stimulates ruminal development. Controls lactic acidosis. Stimulates production of ruminal VFA.	[32]–[34]
	*Propionibacterium*	*P. shermanii*	Increases production of glucose (gluconeogenesis). Spares glucogenic amino acids. Inhibits hepatic lipid oxidation. Reduces CH_4_ production. Impacts feed intake and feed efficiency. Increases ruminal synthesis of propionate. Increases weight gain and feed efficiency. Decreases the incidence of the metabolic disorders like acidosis and ketosis.	[35],[36]
		*P. freudenreichii*		
		*P. acidipropionici*		
		*P. jensenii*		
Other bacteria	*Prevotella*	*P. bryantii*	Diverts the hydrogen flow in glycolysis away from methanogenesis. Favors propionic acid production. Improves lignocellulose processing. Competes with methanogenesis and archaea for hydrogen utilization. Reduces CH_4_ emissions.	[36]–[38]
	*Bacillus*	*B. subtilis*	Supports adequate health, nutrient digestibility. Improves performance of lactating dairy cows. Improves milk lactose yield, and total solids yield of lactating dairy cows. Changes rumen fermentation and bacterial profiles. Improves growth performance. Stimulates GH/IGF-1, and regulates the gut microbiota. Produces active dietary enzymes such as amylase, lipase, phytase and protease.	[19],[21],[39]–[43]
		*B. licheniformis*		
		*B. coagulans*		
Fungi	*Saccharomyces*	*S. cerevisiae*	Reduces CH_4_ production. Enhances nutrient digestion. Induces inflammatory responses. Increases fiber digestion. Increases initial rates of fiber digestion. Increase milk production in dairy cows. Improves ruminal microbial activities. Increases the number of total anaerobic and cellulolytic bacteria. Stimulates lactate uptake. Provides important nutrients and nutritional cofactors that stimulate microbial activities. Provides vitamins such as biotin and thiamine.	[30],[44]–[50]
		*S. boulardii*		
Fungi	*Aspergillus*	*A. oryzae*	Improved the feed dry matter digestibility. Improves energy supply of VFA Concentrations. Powerful antioxidative functions. Contains multiple enzymes, especially cellulase and hemicellulose. Improves the fiber type in feed. Enhances nutrient absorption. Produces phytate.	[51]–[53]
		*A. niger*		

LAB = lactic acid-producing bacteria, LUB, Lactic acid utilizing bacteria.

The preferred time to use microbial feed additives is when animals are under environmental stress (thermal, moisture, crowding, and sanitary conditions), emotional stress (handling or shipping, changes in pen-mates, and weaning), and disease stress (nutrient shortage or excess, and antagonism between levels of two or more nutrients) to help in establishing a beneficial microorganism population in the digestive system to reduce or prevent harmful organism establishment [3]. The mode of action of bacterial DFM are sensitive to some factors including doses, feeding periods and frequencies, and strains, with different modes of actions between the rumen and the gastrointestinal system [4]. Within the rumen, LAB avoids ruminal acidosis in dairy animals [33],[54]. Lactic acid utilizing bacteria facilitate the utilization of lactic acid while maintaining ruminal pH in the rumen [54].

Feeding a highly fermentable diet supplemented with *Megasphaera elsdenii* (the predominant lactate-utilizing bacteria in the rumen), prevented the severe pH reductions induced by lactate production in the rumen [55]. Another result observed with feeding probiotic on ruminal environment is increasing the production of propionate and numbers of propionic-producing bacteria [41]. Increasing propionate enhances hepatic glucose production, increases lactose synthesis, improves energy efficiency, reduces ruminal ketosis, and reduces methane (CH_4_) generation [56],[57].

In the post-ruminal gastrointestinal tract, feeding microbes restricts and prevents the growth and activity of pathogens from sticking to the intestinal mucosa via hydrophobic interactions, as well as limit pathogens from binding to the enterocytic receptor or creating enterotoxins that cause diarrhea [58]. Lactic acid bacteria administration was able to stick to the intestine and protect the mice against Salmonella [59]. Moreover, LAB forms chemicals substances with antibacterial and probiotic properties, such as bacteriocin and hydrogen peroxide that can prevent substrates from binding to the rib nucleotide reductase subunit, therefore interfering with target microorganism DNA synthesis [60]. Another mechanism is the immediate uptake of direct fed microbes by intestinal epithelial cells by transcytosis, which are then engulfed by antigen-presenting cells, macrophages, or dendritic cells, ultimately triggering an immunological response [60].

The most used probiotics bacteria in animal feeding are *Lactobacillus*, *Bifidobacterium*, *Bacillus*, *Enterococcus* and *Lactococcus*.

*Lactobacillus: Lactobacillus* is gram-positive bacteria that is LAB. It includes more than 100 different species, where most of them are a part of the normal mammal's microbiota [22]. Species in this group are commonly introduced as probiotics in both dairy and non-dairy foods intended for human consumption [61],[62]. The *Lactobacillus* strains produce a variety of dietary enzymes including protease amylase, lipase, phytase, and protease which help in digestion and absorption of nutrients [19].

*Bifidobacterium*: *Bifidobacteria* are species of bacteria that are found in the gut of animals and human, where it is considered an indicator of the good health of the host [63]. Bifidobacteria maintains the balance of the intestinal microbiota and limits the risk of infections. It is recognized as “GRAS” (Generally Regarded As Safe) [64]. Bifidobacteria are extensively used as a feed additive to replace the conventional antibiotics, with promising ability to specifically inhibit certain pathogens.

*Bacillus*: *Bacillus* are gram-positive bacteria regularly used as supplements in livestock production. *Bacillus* species possess high potential to modulate the immunity of animals and protect them from diseases [65].

*Enterococcus*: *Enterococcus* is a common member of the endogenous intestinal microbiota of humans and animals [31]. Some species from the genus Enterococcus have been used as probiotic for animals; however, it is not considered as “GRAS” [31].

*Lactococcus*: *Lactococcus* are a group of bacteria that is commonly used in the manufacture of fermented dairy products.

### 2.2. Fungal direct-fed microbes (Yeasts)

Yeast sizes are larger than bacteria and range from 2 to 50 µm in length and 1 to 10 µm in width [66]. In both inner and outer cell walls, β-glucans and mannoprotein are the major components [12]. Moreover, the cell wall of yeast contains a minor component known as chitin, which contributes approximately 1 to 3% in the yeast cell wall, and β-1,6 glucan links to the inner and outer walls, strengthening the cell structure [67]. Interestingly, yeast cell walls could stimulate animals to secrete protease and glucanase to release cell contents and cause fragmentation of cell walls [68].

*Saccharomyces cerevisiae* and *Aspergillus oryzae* are the most commonly employed fungal species utilized to improve performance and regulate rumen fermentation [18],[51]. Saccharomyces is a part of the gut microbiota, that is commonly used as feed additives. Several mechanisms have been proposed to explain changes in ruminal fermentation and improvements in ruminant performance with feeding fungi-based DFM. Fungi administration in ruminants may help the ruminal bacteria (e.g., *Selenomonas ruminantium*) to utilize lactate more effectively by supplying dicarboxylic acids and other growth factors [69]. Additionally, feeding yeast to animal scavenges oxygen from the surfaces of recently eaten feed, allowing the rumen to retain metabolic activity while remaining anaerobic [70]. Lowering the rumen's redox potential, which allows strict anaerobic cellulolytic bacteria to grow more easily, accelerates their adhesion to fodder particles, and boosts the initial rate of cellulolysis was observed with feeding yeast [4]. Another mechanism is the ability of yeast to compete with other starch utilizing bacteria for starch fermentation, limiting lactate buildup in the rumen, supplying growth factors such as organic acids or vitamins, and stimulating ruminal cellulolytic bacteria [71].

### 2.3. Mixed direct-fed microbes

The third major category of DFM is the mixed direct-fed microbes, which combine beneficial bacterial and fungal microorganisms. It could be considered as a valuable tool in modern ruminant nutrition, contributing to improved animal health, productivity, and welfare [72]. As will be detailed later, mixing more than one organism (*i.e*., multi-strain probiotics) in livestock production may cause several symbiotic responses or antagonistic effects [24],[73]. However, the impact of combining multiple probiotics is influenced by the strains chosen, their interactions, and the individual characteristics of the host animal. Proper formulation and dosing are crucial to maximize symbiotic benefits and minimize antagonistic effects, resulting in positive effects on animal performance, in most cases. Emmanuel et al. [30] observed an induced inflammatory response in steers fed high-grain diets supplemented with mixed DFM containing bacteria (*Enterococcus faecium*) and yeast (*S. cerevisiae*).

## 3. Performance of ruminants fed microbes

Tables 2 and 3 show the performance of animals to bacterial- and yeast-based products. Variations between experiments may be due to different animal species, different supplementations, different doses, different stages of production, etc.

### 3.1. Pre ruminant calves

Before rumination, calves can digest a large number of nutrients in their gut; however, this may cause the proliferation of some harmful organisms in their intestine resulting in diarrhea, weight loss or death [45]. In this situation, direct fed microbes may be a good solution to give the calves a rapid ability to be fed and adapted to feeding solid feeds. Direct fed microbes may increase the creation of ruminal and intestinal microbes and prevent the establishment of enteropathogens, which typically results in diarrhea [45]. Administering probiotics to young calves helps them develop the rumen faster [74].

**Table 2.** Effect of bacteria-based products on ruminant performance.

Animal/study design	Diet	Product	Dose	Effects^†^	Reference
Rumen-fistulated dairy cows	F:C was 60:40	*Prevotella bryantii* (25A)	2 × 10^11^ cells/dose per animal daily	↕ Feed intake. ↕ Milk production. ↕ Rumen pH. ↓ Ruminal lactate concentration. ↑ Ruminal concentration of NH_3_-N. ↑ Acetate, butyrate, and branched-chain C_4_ fatty acid concentrations. ↕ Acidosis.	[37]
Holstein cows	Corn and ryegrass silages	Combination of *Lactobacillus acidophilu* NP51 and *Propionibacterium freudenreichii* NP24	4 × 10^9^ CFU/h per day	↕ Feed intake. ↑ Milk yield. ↑ Energy-corrected milk. ↕ Milk fat and protein percentages. ↑ Milk protein yield. ↕ Respiratory rate, skin temperature, body temperature. ↑ Apparent digestibility.	[75]
Male and female Holstein calves	Calf starter feed	*Megasphaera elsdenii*	50 mL oral dose of *M. elsdenii* NCIMB 41125 (10^8^ CFU/mL) per calf daily	↑ Starter DM intake. ↑ Weaning body weight. ↑ Plasma *β*-hydroxybutyrate concentration. ↑ Reticulo-rumen weight. ↑ Papillae width and papillae density. ↕ Total VFA, acetate and propionate production. ↑ Butyrate production.	[34]
Crossbreed of Japanese black cattle and Red Angus cattle	Total mixed ration	*Bacillus amyloliquefaciens* C-1 and *B. subtilis*	4 × 10^10^ CFU/calf daily	↑ Body weight gain. ↑ Feed intake. ↑ Efficient feed conversion rate. ↑ *Proteobacteria, Rhodospirillaceae*, *Campylobacterales*, and *Butyricimonas*. ↓ Pathogens. ↑ *Akkermansia*.	[40]
Murrah buffalo calves	Total mixed ration	*Lactobacillus acidophilus*	Fermented milk containing 200 mL/calf/day (10^8^ CFU/mL)	↑ Final body weight. ↑ DM intake. ↑ Average daily gain. ↑ Feed conversion efficiency. ↑ Digestibility of fiber. ↑ The fecal lactobacilli and bifidobacterium population. ↓ Fecal coliform count. ↑ The fecal VFA.	[20]
Murrah buffalo calves	Total mixed ration	*Lactobacillus acidophilus*	Fermented milk containing 200 mL/calf/day (10^8^ CFU/mL)	↑ Final body weight. ↑ DM intake. ↑ Average daily gain. ↑ Feed conversion efficiency. ↑ Digestibility of fiber. ↑ The fecal lactobacilli and bifidobacterium population. ↓ Fecal coliform count. ↑ The fecal VFA.	[20]
Saanen dairy goats	Total mixed ration	*Bacillus subtilis* and *Enterococcus faecalis*	5 g per goat daily (5 × 1,011 CFU/goat per day)	↑ DM intake. ↑ milk yield. ↑ Protein and lactose percentage with *B. subtilis*. ↕ Bacterial abundance and diversity. ↑ *Succinivibrionaceae*.	[76]
Lactating Friesian cows	F:C was 50:50	*Lactiplantibacillus plantarum*, *Lactobacillus gasseri*, *Leuconostoc mesenteroides* and *Bifidobacterium breve*	20 g/kg silage	↓ Silage contents of oxalic acid and fibers. ↑ Silage content non-structural carbohydrate and calcium. ↕ Milk production. ↑ Energy-corrected milk. ↕ Feed efficiency. ↓ Feed intake. ↓ Total ruminal VFA, acetate and propionate concentrations. ↑ Milk total solids, fat, protein and energy.	[77]
Farafra lambs	F:C was 40:60	*Bacillus subtilis* and *Phanerochaete chrysosporium*	2 and 4 g per lamb daily	↑ Live-weight gain. ↓ Roughage and total feed intake. ↑ Feed efficiency. ↑ Serum total protein, globulin, urea-N, aspartate aminotransferase and alanine aminotransferase. ↑ Hot carcass weight and dressing percentage. ↓ Tail fat and all fat levels.	[39]
Sheep and lambs	Concentrate with freely available bean and cereal hay	*Bacillus subtilis* B-2998D, B-3057D, and *Bacillus licheniformis* B-2999D	1 and 3 g per animal daily	↑ Body weight gain. ↑ Blood total protein, globulins, and urea. ↓ Bilirubin and cholesterol. ↑ Bactericidal and phagocytic index. ↑ Fecal *Lactobacillus* and *Bifidobacterium*. ↓ Fecal *Escherichia coli*, Enterococcus, and yeast.	[43]
Farafra male lambs	F:C was 30:70	2 × 10^11^ CFU of *Bacillus subtilis*/g or 1 × 10^12^ CFU of *Lactobacillus acidophilus*/g or 6 × 10^8^ CFU of *Ruminococcus albus*/g	4 g product per lamb daily	↑ Growth performance and feed efficiency. ↑ Concentrations of albumin, triiodothyronine, thyroxine, hemoglobin and red blood cells. ↓ Globulin and urea-N. ↑ Hot carcass. ↑ *Longissimus dorsi* with *B. subtilis.* ↕ Water holding capacity. ↑ Digestibility of crude protein with *B. subtilis*. ↑ Digestibility of DM, OM, and fiber with *B. subtilis* and *R. albus*. ↕ N intake and urinary N. ↓ Fecal N. ↑ N balance. ↑ Ruminal VFA with *B. subtilis* and *R. albus*.	[78]
Lactating Farafra ewes	F:C at 40:60	*Bacillus subtilis* and *Bacillus lichenifomis*	4 g product/kg DM feed (each g of the product contained 1.75 × 10^12^ CFU *B. subtilis* and 1.75 × 10^12^ CFU *B. lichenifomis*)	↑ Total intake. ↑ Digestibility of all nutrients. ↑ Concentrations of ruminal ammonia ↑ Concentration of total VFA, acetate and propionate. ↑ Milk production. ↑ Concentrations of milk fat, lactose and energy. ↑ Concentrations of total n3, n6 fatty acids, PUFA and CLA. ↓ Aatherogenicity.	[79]
In vitro (Rumen inoculum was collected from sheep)	F:C was 50:50	*Bacillus subtilis* and *Bacillus lichenifomis*	4 g product/kg DM feed (each g of the product contained 1.75 × 10^12^ CFU *B. subtilis* and 1.75 × 10^12^ CFU *B. lichenifomis*)	↓ Asymptotic production of total gas, CH_4_ and CO_2._ ↓ Rate of CH_4_ and CO_2_. ↑ Lag time of CH_4_ and CO_2_ production. ↑ Total bacterial count. ↑ Fermentation pH and VFA. ↓ Protozoal count.	[80]
Ongole breed cattle		*Lactiplantibacillus plantarum* (1.8 × 10^10^ CFU/mL)	10, 20 and 30 mL/cow daily	↓ Ruminal acetate. ↑ Ruminal propionate. ↓ Ruminal protozoal number. ↕ Ruminal *L. plantarum.* ↕ Ruminal *Ruminococcus flavefaciens.* ↕ Ruminal *Treponema bryantii.* ↑ Ruminal *Ruminococcus albus*.	[41]
A meta-analysis study	Different diets	*Megasphaera elsdenii*	Different doses	↑ Ruminal propionate, butyrate, isobutyrate, and valerate. ↓ Ruminal lactic acid concentration, acetate proportion. ↓ Ruminal total bacterial population. ↓ CH_4_ emission. ↑ Average daily gain and body condition score. ↑ Carcass quality. ↑ Hot carcass weight. ↑ Carcass gain. ↓ Diarrhea, bloat incidences and liver abscess.	[33]

CFU = colony forming unit, CLA = conjugated linoleic acids, DM = dry matter, F:C = Forage:concentrate ratio, PUFA = polyunsaturated fatty acids, VFA = volatile fatty acids, UFA = unsaturated fatty acids, ↑ = increased, ↓ = decreased, ↕ = no effect. ^†^Effect is relative to control.

**Table 3.** Effect of fungi-based products on ruminant performance.

Animal/study design	Diet	Product	Dose	Effects^†^	Reference
Awassi lambs and Shami goat kids	High concentrate diet	*S. cerevisiae*	12.6 g yeast/ton of diet	↕ Growth performance of lambs and kids. ↑ Fat content in the carcass. ↕ Digestibility of DM, CP and NDF. ↑ Digestibility of OM and ADF. ↕ N intake, output or retention. ↕ DM intake. ↓ Hot carcass weight, cold dressing proportion and total muscle/bone ratio in lambs. ↑ Empty digestive tract weight in lambs. ↑ Digestibility of nutrient in kids. ↕ Growth, feed intake or feed conversion ratio of fattening lambs and kids.	[81]
Multiparous Holstein cows	F:C at 60:40	Active dry *S. cerevisiae* yeast	0.5 g of active dry yeast/cow daily (10^10^ CFU daily)	↑ Ruminal pH. ↑ Ruminal butyrate concentration. ↕ DM intake. ↕ Ruminal ammonia-N. ↕ Ruminal VFA.	[50]
Rambouillet lambs	Forage and concentrate	*S. cerevisiae*	0.25 and 0.35 mg Cr-yeast or 0.3 mg Se-yeast/lamb daily	↓ Fat in the carcass. ↑ Average daily weight gain. ↑ Final body weight. ↕ Daily weight gain and total weight gain. ↕ Feed intake, and feed conversion.	[82]
Crossbred young bulls (Zebu × European)	F:C at 44:56	*S. cerevisiae*	15 g of yeast per bull daily	↑ Linolenic acid concentration in meat. ↓ n6:n3 ratio in meat. ↑ α-linolenic fatty acid. ↑ α-linolenic, arachidonic, eicosapentaenoic, docosapentaenoic, docosahexaenoic and n-3 fatty acids in meat. ↕ SFA, poly UFA, n-6 fatty acids and n-3.	[83]
Lambs	High concentrate diet	Live yeast cultures (*Kluyveromyces marximanus*, *S. cerevisiae*, *Saccharomyces uvarum*)	1.5×10^9^ to 2.0 × 10^9^ CFU per kg live weight	↑ Ciliate protozoa. ↑ Feed intake. ↑ Growth rate. ↑ *Entodinomorphs* population. ↕ Half carcass weight. ↕ Carcass traits. ↓ Fluid pH and total VFA with concentration with *S. uvarum*. ↑ Ruminal Diplodinomorphs population with *S. uvarum*. ↑ Proteases activity ↓ α-amylase activity with *K. marximanus* and *S. cerevisiae*. ↕ Carboxymethyle cellulase activity.	[84]
Kamieniecka lambs	Hay silage and concentrate	*S. cerevisiae*	50 g/kg concentrate daily	↑ Protein content. ↓ Cooking loss. ↑ Water-holding capacity of meat. ↑ *cis-*9, *trans-*11 CLA, C14:1, C18:2 and C22:6 fatty acids in the intramuscular fat.	[85]
Kamieniec rams	Forage and concentrate	*S. cerevisiae*	50 g yeast/kg of diet	↑ Concentration of C14:1, C18:2, C22:6, C18:2 (*cis*-9 *trans*-11) in meat. ↑ Vitamin A content of meat. ↑ Intramuscular fat. ↑ Fat, cholesterol, and vitamin E of meat.	[86]
Santa Ines lambs	F:C at 40:60	Inactive dry *S. cerevisiae* yeast	Inactive dry yeast at 4.87, 9.73, and 14.60% of diets	↕ Ruminal pH ↓ The subcutaneous fat thickness. ↑ Meat crude protein and ash. ↕ Growth performance.	[87]
Holstein Friesian cows	Concentrate	*S. cerevisiae*	2.5 g yeast/cow daily (2.5 10^10^ CFU daily)	↑ Milk yield. ↕ Milk composition. ↕ Milk pH.	[88]
Finnish Ayrshire cows	F:C at 50:50	*S. cerevisiae*	0.5 g live yeast/cow daily (10^10^ CFU/cow daily)	↕ Animal performance. ↕ Rumen fermentation. ↕ Milk yield. ↕ Milk composition. ↕ Milk fatty acids. ↕ DM intake. ↕ Ruminal gas production. ↕ Apparent total-tract nutrient digestibility. ↕ Ruminal CH_4_ emission.	[46]
In vitro (Rumen inoculum was collected from Brown Swiss cows)	F:C at 50:50	Live cells or cells extract of *S. cerevisiae*	Live cells at 0.3, 0.6 and 0.9 mg/g DM or cells extract at 1, 2 and 4 mg/g	↑ Asymptotic gas production with the extract. ↕ DM degradability. ↑ CH4, metabolizable energy and total VFA with cell extract.	[89]
Barki lambs	F:C at 70:30	*Trichoderma reesei* or *S. cerevisiae*	0.5 g/kg DM feed	↓ Feed intake. ↑ Feed conversion efficiency. ↑ Ruminal VFA. ↑ Blood albumin and urea concentrations. ↑ Dressing percentage. ↓ Intramuscular fat weights. ↕ Histology of the ileum, sub mucosa and Peyer's patches.	[44]
Pelibuey sheep	Total mixed ration	Selenium-enriched *S. cerevisiae* yeast	0.35 and 0.60 mg Se/kg DM	↕ Microbiological variables (aerobic plate counts, total coliform counts and fecal coliform counts). ↕ Carcasses microbial growth. ↕ Carcasses initial and ultimate pH. ↕ Carcasses temperature, color values. ↕ Carcasses water holding capacity.	[90]
Simmental × Luxi F1 crossbred bulls	Forage and concentrate	*S. cerevisiae* active dry yeasts and yeast cultures	0.8 g dry yeast/bull daily and 50 g yeast culture/bull daily	↕ Intramuscular fat content. ↕ Cholesterol content. ↓ Backfat thickness. ↑ Concentration of free fatty acids in the blood. ↑ Growth performance. ↑ Carcass traits. ↑ Beef tenderness.	[91]
Holstein bull calves in the first 56 d of life	Milk and starter grains (no forage)	*S. cerevisiae* fermentation products	0.5% and 1% in starter diets and milk	↑ Ruminal *Butyrivibrio.* ↓ Ruminal *Prevotella*. ↑ Ruminal butyrate concentration. ↕ Ruminal pH, ammonia-N, and total VFA. ↑ Papilla length in the rumen. ↓ Crypt depth of jejunum. ↑ Villus height-to-crypt depth ratio in the small intestine.	[92]
Rumen-fistulated Holstein dairy cows	F:C at 40:60	Active dried *S. cerevisiae*	10 g yeast/cow daily (20 × 10^9^ CFU/cow daily)	↑ *Fibrobacter succinogenes*. ↑ *Megasphaera elsdenii.* ↕ DM intake. ↕ Ruminal pH characteristics. ↕ Ruminal ammonia-N. ↑ Blood glucose and insulin. ↓ Ruminal protozoa.	[93]
Crossbred Friesian × Baladi calves	Starter concentrate feed	*S. cerevisiae*	2.5 and 5 g yeast per calf daily.	↑ Feed intake with the high level. ↑ DM and fiber digestibilities. ↓ Ruminal ammonia-N. ↑ Total and individual VFA. ↑ Final weight and daily gain. ↓ *Clostridium* spp., *Escherichia coli* and *Enterobacteria* spp.	[18]
In vitro (Rumen inoculum was collected from sheep)	Two mixed rations with two different levels of CP of 13% and 16% on DM basis	*S. cerevisiae*	2 and 4 mg/g DM	↑ The asymptotic gas production. ↓ Lag time of gas production. ↕ Rate of GP. ↕ Gas yield at 24 h of incubation, microbial crude protein production, metabolizable energy, partitioning factor at 24 h of incubation. ↕ DM and OM degradability.	[94]
In vitro (Rumen inoculum was collected from sheep)	Total mixed rations	*S. cerevisiae*	2 and 4 mg/g of DM	↓ Asymptotic gas production. ↓ CH_4_ production. ↑ CO_2_ production. ↕ DM degradability.	[48]
Holstein cows	Grass silage and concentrate	*S. cerevisiae*	0.8 g yeast/cow daily (1 × 10^10^ CFU/cow daily) and 4.0 g yeast/cow daily (6 × 10^10^ CFU/cow daily)	↕ Milk yield. ↕ Milk fat ↕ Milk protein. ↕ Milk lactose.	[95]
Nubian lactating does	F:C at 50:50	*S. cerevisiae*	4 g yeast/doe daily	↑ Feed intake and feed (milk) efficiency. ↑ Milk and energy corrected milk yields. ↑ Concentrations of milk total solids, solids-not-fat, fat, and lactose. ↑ Nutrient digestibility. ↑ Ruminal pH. ↑ Concentrations of ruminal total and individual VFA. ↓ Ruminal ammonia-N. ↑ Blood total proteins, albumin, globulin, and glucose.	[96]
Holstein cows	Forage and concentrate	Yeast *S. cerevisiae* culture	15 g inactivated dry yeast/cow daily	↑ Milk yield. ↑ Milk fat and lactose concentrations. ↑ Ruminal pH. ↑ Feed efficiency. ↕ DM intake. ↑ Ruminal propionate. ↓ Ruminal ammonia-N. ↑ Microbial N synthesis.	[97]
Malpura lambs	High starch diet (no forage)	*S. cerevisiae*	Yeast at 9.0 × 10^7^ CFU/kg BW	↕ pH. ↕ Carcass characteristics. ↑ Digestibility of ADF.	[47]
Charolais bulls	High concentrate diet	Live yeast of *S. cerevisiae* CNCM I-1077	5 g yeast/bull or (1 × 10^10^ CFU/bull daily)	↕ Ruminal pH. ↑ Acetate and butyrate concentrations. ↑ Acetate:propionate ratio. ↕ Final bodyweight. ↕ Average daily gain. ↑ DM intake. ↕ Carcass weights and dressing. ↑ Carcass graded.	[98]
Dry Holstein cows	F:C at 70:30	Live yeast of *S. cerevisiae*	3.3 g yeast/kg of diet daily (1 × 10^10^ CFU/d)	↑ *Bacteroidales*. ↑ *Lachnospiracea*. ↑ *Flexilinea*.	[99]
Holstein steers	F:C at 50:50	*S. cerevisiae*	Live yeast at 15 g/d	↑ *Ruminococcus albus.* ↑ *Ruminococcus champanellensis.* ↑ *Ruminococcus bromii.* ↑ *Ruminococcus obeum.* ↑ *Megasphaera elsdenii.* ↑ *Desulfovibrio desulfuricans.* ↑ *Nitratidesulfovibrio vulgaris*.	[70]
Small-tailed Han lambs	Pelleted total mixed rations	*S. cerevisiae* yeast culture	0.8 and 2.3 g of yeast/kg dietary feed	↑ Linoleic acid concentration in the muscle. ↓ Conversion of linoleic acid to stearic acid. ↑ DM intake. ↑ Carcass weight and dressing percentage. ↕ Growth performance. ↕ Carcass traits.	[100]
Holstein cows	Forage and concentrate	Live *S. cerevisiae* yeast	5.4 × 10^11^ CFU yeast daily	↕ Milk yield. ↕ Milk fat, lactose and protein yields. ↕ Milk fat, protein and lactose concentration. ↕ DM intake. ↕ body weight gain. ↓ DM digestibility. ↑ CP, NDF and starch digestibility.	[101]
Holstein cows	Forage and concentrate	*S. cerevisiae*	4 g yeast/cow daily	↑ Milk yield. ↑ Milk total solid. ↑ Milk fat concentration. ↑ Prepartum DM intake. ↕ After parturition DM intake. ↕ Loss of body condition score from calving to d 21 postpartum. ↕ Glucose tolerance test. ↕ Cellular immune function.	[49]
Qinchuan cattle	F:C at 55:45	*S. cerevisiae*	1 and 2 g live yeast or 20 g yeast cell wall polysaccharides/cow daily	↑ Digestibility of ADF and NDF. ↑ *Fibrobacter succinogenes* S85. ↑ *Ruminococcus albus.* ↑ *Ruminococcus flavefaciens* FD-1. ↓ *Streptococcus bovis* JB1. ↑ Average daily gain.	
Qinchuan cattle	F:C at 55:45	*S. cerevisiae*	1 and 2 g live yeast or 20 g yeast cell wall polysaccharides/cow daily	↓ Feed conversion ratio. ↑ Digestibility of NDF and ADF. ↓ Acetic:propionic ratio.	[102]
Rambouillet ram lambs	Concentrates	Chromium enriched *S. cerevisiae* yeast	0.3 mg Cr enriched yeast/kg feed	↑ Backfat thickness. ↑ Meat pH. ↕ Fatty acid profile of meat.	[103]
Sohagi ewes	Concentrate and forage	Active dry *S. cerevisiae*	5 or 10 g of yeast per ewe daily	↑ Milk yield. ↑ Milk fat. ↑ Milk protein. ↑ Milk solids non-fats. ↕ Milk lactose and ash. ↑ Blood total protein, albumin, glucose and urea. ↕ Blood globulin, cholesterol, creatinine and alanine aminotransferase concentrations.	[104]
Lactating Holstein cows	F:C at 408:592	*S. cerevisiae* and/or *Aspergillus oryzae*	3.5 g per cow daily	↑ Feed intake. ↑ Milk production. ↓ Milk fat content with *A. oryzae*. ↓ Serum glucose concentration.	[51]
Beef cattle	A meta-analysis	*S. cerevisiae*	A meta-analysis	↑ Average daily gain. ↑ Carcass weight. ↑ Final body weight.	[105]
Sheep	Concentrates	*S. cerevisiae*	0.4, 0.8 and 1.2% (w/w) to concentrate	↑ Ruminal enzymatic activity. ↑ Rumen microbiota. ↑ Animal growth intensity. ↑ Production of total nitrogen and amylolytic, proteolytic and cellulolytic activity of the rumen microbiota.	[106]

ADF = acid detergent fiber; CFU = colony forming unit; CH_4_ = methane; CO_2_ = carbon dioxide; CP = crude protein; DM = dry matter; F:C = Forage:concentrate ratio; NDF = neutral detergent fiber; OM = organic matter; VFA = volatile fatty acids; SFA = saturated fatty acids; UFA = unsaturated fatty acids; ↑ = increased; ↓ = decreased; ↕ = no effect; ^†^Effect is relative to control.

### 3.2. Adult ruminants

Administration of microorganisms is more common in ruminant animals with a mature rumen, especially those with high milk production since they are in a negative energy balance and fed diets rich in fermentable carbohydrates [69]. Probiotics have demonstrated their ability to strengthen rumen fermentation, decrease zoonotic pathogens and control ammonia production. Chen et al. [42],[107] observed that *Rhodopseudomonas palustris* supplementation promoted the viability of rumen microorganisms, rendered high growth performance of rumen microorganisms and increased microbial fermentation to keep up the microbial balance.

During the transition periods, cows may be subjected to many metabolic disorders such as calving stress, changing diets to rapidly fermented carbohydrate sources, and lactation. Feeding microbials was employed to improve the performance of lactating animals by boosting intake, milk production, milk protein content, and pre-and post-partum blood glucose and insulin levels [26],[108]. The mature rumen has an intricate microbial population. Ruminal microbes ingest and degrade carbohydrates and proteins. *S. cerevisiae* [51],[96], LAB [77],[79],[80], *Aspergillus oryzae* [51], *Bacillus* and *Enterococcus* [39],[76] are the commonly used probiotics in ruminants.

Feeding live microorganisms enhanced milk production in dairy cattle. *Bacillus subtilis*, *S. cerevisiae*, and *Enterococcus faecalis* increased milk secretion [51],[76],[96]. Moreover, feeding probiotics increased the animal growth performance [39],[40],[78],[109]. *Bacillus subtilis* and *Bacillus amyloliquefaciens* upgraded the intestinal maturation and growth competency by stimulating GH/IGF-1 hormone [40]. Probiotics can enhance the immunity in ruminants. *Lactobacillus acidophilus*, *Ligilactobacillus salivarius*, and *Lactiplantibacillus plantarum* at 10^7^–10^8^ CFU/g lowered the occurrence of diarrhea in juvenile calves [110].

## 4. Practical considerations of feeding microbial feed additives

Microbial commercial products have to be formulated to be stable in heat and can withstand low pH conditions in the stomach, are cheap commercially, have a long shelf life, are stable in the feed, and can withstand the process of heat palletization [6].

Microbial based feed additives are presented in many forms, including powders, pastes, boluses, and capsules. Microbial feed additives may be mixed with feed or injected into drinking water; however, injecting into water is not preferred due to probable interactions with chlorine, water temperature, minerals, flow rate, and antibiotics [69]. Non-hydroscopic whey, vegetable oil, and inert gelling agents are frequently employed as carriers for bacterial feed additives, while grain by-products are the carriers for fungal based feed additives.

Some microbial based feed additives are made for one-time use, while others are intended to be fed regularly. The doses of microbial feed additives vary from product to another one. However, 10^6^ to 10^10^ CFU per animal daily is the common range for most bacterial feed additives.

One of the major problems that should be considered during the administration of microbial feed additives during feed pelleting is their sensitivity to heat. Therefore, heat tolerance of microorganisms is critical because heat may kill the majority of yeast, *Lactobacillus*, *Bifidobacterium*, and *Streptococcus*. However, increasing the level of microbial fed additives may compensate for microbial loss during pelleting. Microbial feed additive products should be maintained free from dampness, excessive heat, and light, and sometimes oxygen.

## 5. Symbiotic and antagonistic effects between microbial feed additives

Using multistrain probiotics in livestock production is a common practice. However, combining many probiotics together before feeding them to animals may cause several symbiotic responses or antagonistic effects [24],[73]. The symbiotic responses between more than one probiotic include: (1) Enhancing gut health through enhancing the balance of gut microbiota, enhancing nutrient absorption and overall gut health, (2) increasing the production of fermentation beneficial metabolites such as VFA, and (3) improving digestive enzyme production and activity resulting in improving the efficiency of digestion and nutrient utilization [111]. Such effects will promote better performance in animals either as milk or meat production [24].

Sometimes, combining more than one probiotic could result in antagonistic effects such as increased competition between microbes for the same nutrients and adhesion sites in the gut, potentially reducing the effectiveness of one or more strains, and the production of some substances that inhibit the growth of other strains, which may reduce the overall probiotic effect [24]. Moreover, antagonistic effects include unbalancing the gut microbiota and disrupting the natural balance of gut microbiota, potentially leading to dysbiosis [24],[112]. Overstimulation of the immune system is another possible antagonistic effect, potentially leading to inflammation or other adverse effects [7].

Generally, the effects of combining multiple probiotics depend on the specific strains used, their interactions, and the host animal's individual characteristics. Proper formulation and dosing are essential to maximize symbiotic benefits and minimize antagonistic effects. Moreover, there is always a potential for some microorganisms from probiotics to overpopulate or become pathogenic under certain conditions; however, probiotics are generally considered safe and beneficial for most animals when used appropriately.

## 6. Future research directions and challenges

Using probiotic feed additives has some future research directions and challenges. The use of probiotics in animal diets is an area of active research with several promising future directions and notable challenges. The future research directions include investigating the specific effects of different probiotic strains on various animal species and understanding the mechanisms by which specific strains impact health and productivity. Optimized probiotic formulations that combine multiple strains for synergistic effects, and studying how probiotics influence the gut microbiome composition and function is another important future research direction. Additionally, using metagenomics and other advanced techniques to profile microbiome changes and link them to health outcomes is another important research direction. Researchers should focus on identifying probiotic strains that can reduce the need for antibiotics by boosting natural immunity, and understanding how probiotics can improve nutrient absorption and utilization.

Moreover, there are many challenges facing the use of probiotic feed additives such as strain variability and consistency and reliability of probiotic strains across different batches and products, as well as addressing variability in strain effectiveness due to differences in animal species, breeds, physiological status and individual health conditions. Determining optimal dosages and delivery methods for different animal species and production systems, and developing stable formulations that maintain viability and activity during storage and administration, with balancing the costs of probiotic production and application with economic benefits for farmers. Conducting long-term studies to assess the sustainability and benefits of probiotic use is another challenge. Understanding complex host-microbe interactions and how they influence probiotic efficacy cannot be ignored as an important challenge for administering microbial feed additives in the diets of ruminants. By addressing these challenges and pursuing the outlined research directions, the potential of probiotics in animal diets can be more fully realized, leading to healthier animals, improved productivity, and more sustainable agricultural practices.

## 7. Conclusions

Using microorganisms from fermentation is one of the sustainable solutions for the environmental challenges of animal production, increasing feed quality, improving animal performance, and animal health. Selected microorganisms can reduce the use of traditional feed additives and can be considered as promising alternatives for antimicrobials. It can be used both pre- and after-rumination. Feeding microorganisms affects rumen and gastrointestinal tract including the production of organic acids, antimicrobial production, competitive exclusion, immunological stimulation, enzyme activity, and toxic amine reduction. Microbial feed additives may be administered as a supplement in feed or drinking water.

Choosing a particular type of microbial feed additive is influenced by numerous factors, such as the strain of microorganism, the species of animal, the type of production (milk or meat), the physiological condition of the animal, genetic factors of the host, diet, age, existing gut microbiota composition, and management practices. The administration of microbial feed additives has many future research and development directions. Therefore, research to investigate the specific impacts of diverse probiotic strains on various animal species and elucidating the mechanisms through which these strains affect health and productivity is a critical issue. Optimizing probiotic formulations by combining multiple strains for synergistic benefits is crucial, as well as studying their influence on gut microbiome composition and function using advanced omics techniques should gain more traction. Therefore, more research should be done to standardize the use of specific microorganisms in livestock production.

## Use of AI tools declaration

The authors declare they have not used Artificial Intelligence (AI) tools in the creation of this article.
